# On Calculation of the Electrostatic Potential of a Phosphatidylinositol Phosphate-Containing Phosphatidylcholine Lipid Membrane Accounting for Membrane Dynamics

**DOI:** 10.1371/journal.pone.0104778

**Published:** 2014-08-20

**Authors:** Jonathan C. Fuller, Michael Martinez, Rebecca C. Wade

**Affiliations:** 1 Molecular and Cellular Modeling Group, Heidelberg Institute for Theoretical Studies, Heidelberg, Germany; 2 Zentrum für Molekulare Biologie der Universität Heidelberg (ZMBH), DKFZ-ZMBH Alliance, Heidelberg, Germany; University of Cambridge, United Kingdom

## Abstract

Many signaling events require the binding of cytoplasmic proteins to cell membranes by recognition of specific charged lipids, such as phosphoinositol-phosphates. As a model for a protein-membrane binding site, we consider one charged phosphoinositol phosphate (PtdIns(3)P) embedded in a phosphatidylcholine bilayer. As the protein-membrane binding is driven by electrostatic interactions, continuum solvent models require an accurate representation of the electrostatic potential of the phosphoinositol phosphate-containing membrane. We computed and analyzed the electrostatic potentials of snapshots taken at regular intervals from molecular dynamics simulations of the bilayer. We observe considerable variation in the electrostatic potential of the bilayer both along a single simulation and between simulations performed with the GAFF or CHARMM c36 force fields. However, we find that the choice of GAFF or CHARMM c36 parameters has little effect on the electrostatic potential of a given configuration of the bilayer with a PtdIns(3)P embedded in it. From our results, we propose a remedian averaging method for calculating the electrostatic potential of a membrane system that is suitable for simulations of protein-membrane binding with a continuum solvent model.

## Introduction

Cell membranes consist of phospholipid bilayers with proteins and other molecules embedded in them. Phosphatidylcholine lipids are neutral dipolar phospholipids that account for up to 60% of total membrane phospholipid composition, with the percentage varying between different cellular membranes and between organisms [1]. Specific binding of proteins to lipids in a bilayer can be mediated by charged phosphatidylinositol lipids which are present in differing concentrations and in a variety of cellular compartments [2], [3]. The electrostatic properties of the phosphatidylinositol lipids and the surrounding lipids in the bilayer are drivers of the protein-lipid association process. Biological membranes are highly dynamic. Here we address the problem of computing the electrostatic potential of a phosphatidylcholine lipid bilayer with an embedded phosphatidylinositol phosphate under consideration of the dynamic nature of the bilayer.

Phosphatidylinositol lipids have been shown to behave as cellular signposts, with specific proteins recognizing phosphatidylinositol lipids that are differently phosphorylated [3], [4]. Examples of protein domains shown to bind phosphatidylinositol lipids are PH, PX, FYVE, PROPPIN, ENTH, and ANTH [3]. The recognition of phosphoinositol-phosphate lipids by binding proteins has been studied computationally. In particular, Lumb *et al*. performed molecular dynamics (MD) simulations of the GRP1-PH domain bound to PtdIns(3,4,5)P_3_ in a 1-palmitoyl-2-oleoyl-sn-glycero-3-phosphocholine (POPC) bilayer using the GROMOS43A1 force field and custom parameters for the PtdIns(3,4,5)P_3_ molecule [5]. Blatner *et al*. investigated the electrostatic properties of several FYVE domains known to bind to PtdIns(3)P [6], showing that mutants with a region of reduced positive electrostatic potential have lower affinities and association rates for binding a PtdIns(3)P containing membrane.

To calculate kinetic parameters for macromolecular diffusional association and gain insight into the factors governing molecular recognition, one can use Brownian dynamics (BD) simulations. To perform BD simulations of a protein-lipid membrane interaction, an accurate continuum solvent representation of the electrostatic potential of both interacting entities is required. One of the most accurate approaches is to solve the Poisson-Boltzmann (PB) equation numerically. This approach is widely used to calculate protein electrostatic potential [7]–[9]. The electrostatic potential can be used for a variety of purposes, including, but not limited to, free energy calculations, visualization, quantitative comparison or BD simulations. The PB equation has also been used to compute the electrostatic potentials of lipid bilayers [10] and proteins embedded in lipid membrane [10], [11]. However, given that lipid bilayers are inherently more dynamic than most proteins, the question arises of which lipid bilayer structures are suitable for computing electrostatic potentials for studying molecular binding to membranes by BD simulation or other continuum solvent methods.

The electrostatic potential of a POPC lipid membrane has been calculated by Li *et al.* from an idealised regular bilayer and also from snapshots taken from an MD trajectory [12]. Li *et al.* also simulated POPC bilayers containing PtdIns(4,5)P_2_ and PtdIns(3,4,5)P_3_ [12]. They found that using either a regular bilayer structure or a snapshot from an MD simulation resulted in a −0.6 kcal/mol/e (−25 mV/kT/e) equipotential bulge in agreement to two standard errors of the mean equipotential bulge height. Lupyan *et al.*, on the other hand, showed that introducing a single PtdIns(4,5)P_2_ into a DPPC bilayer simulated using the CHARMM 27 force field parameters introduces a local perturbation into the lipid bilayer [13]. Conceptually, it is difficult to reconcile these two studies, which use different lipids and different ensembles, since local changes in the bilayer arrangement seem likely to result in changes in the electrostatic potential in the vicinity of these rearrangements. There is still uncertainty about the structure and behavior of these bilayers. Furthermore, repeated biochemical measurements taken over a range of biologically relevant temperatures and experimental conditions, that would allow for better calibration and testing of bilayer simulations, are not routinely made [14]. However, understanding bilayer behavior in regions containing phosphatidylinositol lipids is critical for understanding the structural and energetic basis of many important biological processes which require proteins to associate with phosphatidylinositol containing bilayers to perform their function.

The time-step used in MD simulations is typically about 1 or 2 femtoseconds, whereas molecular association to lipid bilayers occurs on times of nanoseconds to microseconds and beyond. This discrepancy between time-scales means that the electrostatic properties calculated from a single snapshot taken from MD trajectories may not be directly relevant for studying association processes. To begin to address the problem of differing timescales, we developed an ‘effective’ electrostatic potential that may be used in BD studies of protein-lipid-membrane interactions. To calculate the ‘effective’ potential, we use the remedian, a memory efficient statistical method similar to the median, to calculate an effective electrostatic potential that uses information from individual snapshots from an MD simulation, but is relevant over the picosecond-microsecond scales over which molecular associations can occur. We investigated this approach with two lipid force fields, the General Amber Force Field (GAFF) and CHARMM c36 [15], [16], which were chosen as they had been previously used for POPC bilayers and were designed to be compatible with protein and small-molecule force fields (AMBER [17], [18] and CHARMM [16], respectively).

## Results and Discussion

Differences in partial charges in the GAFF and CHARMM c36 force fields do not significantly alter the height of the PtdIns(3)P isopotential bulge. Li *et al.* calculated the height of the −0.6 kcal/mol/e electrostatic isopotential bulge above PtdIns(4,5)P_2_ and PtdIns(3,4,5)P_3_ lipids embedded in a POPC membrane [12]. The electrostatic potential for a ‘regular’ membrane structure is defined by placing lipid molecules on a hexagonally repeating lattice as detailed in the methods. Separately using the GAFF and the CHARMM c36 force field parameters, we found that there is no significant difference in the height of the −0.6 kcal/mol/e isopotential bulge above the PtdIns(3)P. The bulge reaches 13.35 Å above the P1 phosphate of the PtdIns(3)P when the GAFF force field parameters are used, and 13.50 Å when the CHARMM c36 parameters are used. This indicates that the charge parameters for both force fields result in similar electrostatic potentials beyond the −0.6 kcal/mol/e isopotential bulge, when the same set of atomic coordinates is used.

**Figure 1:**
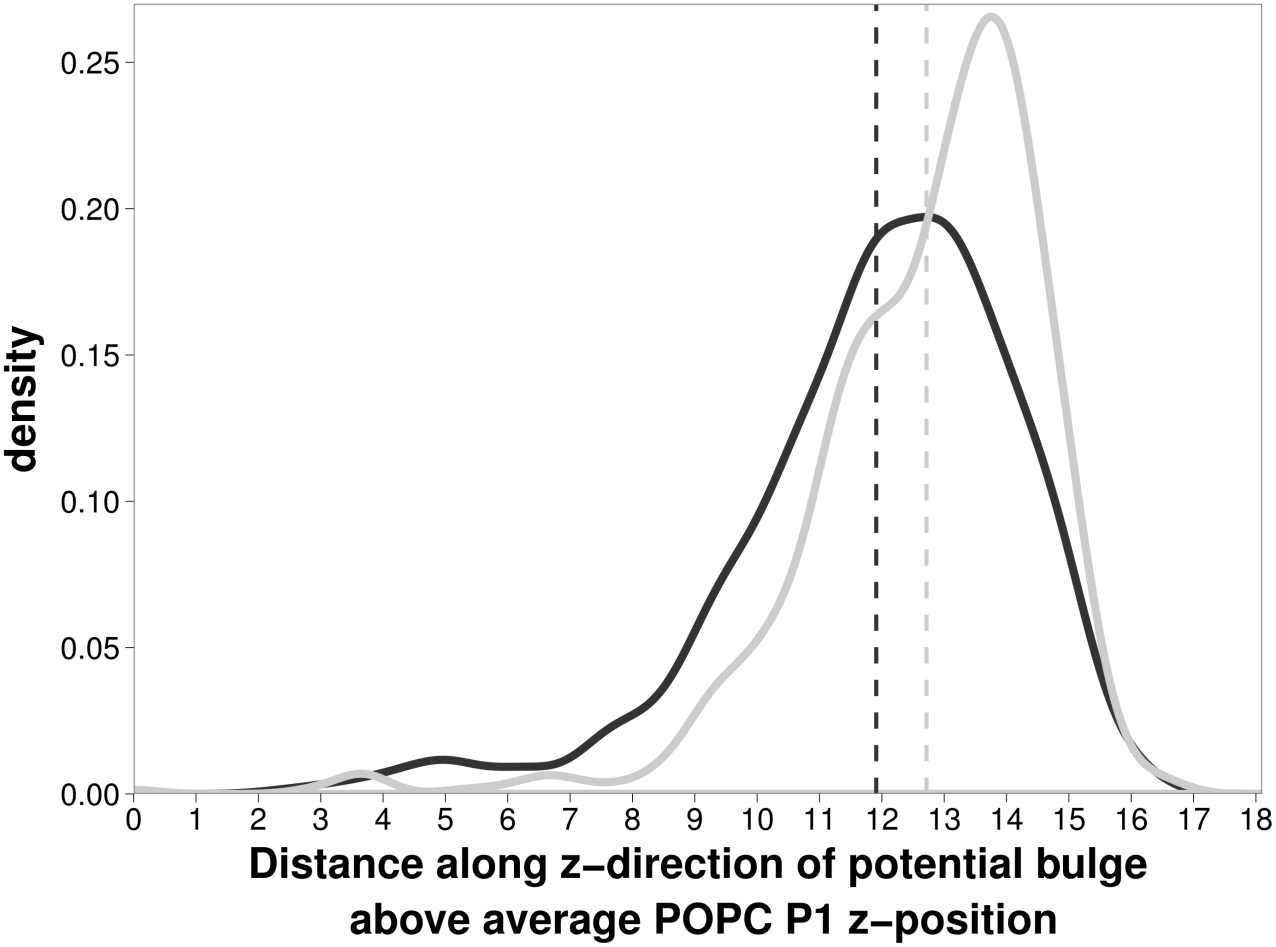
Distribution in the z-direction perpendicular to the bilayer of the bulge in the electrostatic potential. Distances are measured relative to the POPC phosphate positions for the time interval from 30 ns to 100 ns. The distribution shows that the mean z-distance of the bulge in potential for the CHARMM c36 simulation is 0.8 Å higher (12.7 Å) (light gray line), compared to GAFF simulations (11.9 Å) (dark gray line).

Force field choice does not alter the isopotential bulge, but the choice of membrane representation does. In the previous analysis, the same ‘regular’ membrane structure was used, by performing a similar analysis of the height of the isopotential bulge for different snapshots taken from the GAFF and CHARMM c36 simulations, we observed a statistically significant deviation in the isopotential height, with the CHARMM c36 isopotential bulge extending on average 1 Å further into the solvent than the GAFF isopotential bulge. Figure 1 shows that the distribution of the isopotential bulge heights observed during the MD trajectory is narrower for the CHARMM c36 simulations than the GAFF simulations. Given that the force field choice makes no difference for the same membrane structure, the variation in isopotential height for different lipid configurations indicates that the treatment of membrane flexibility is likely to have an important role when considering properties that rely on electrostatic calculations such as the determination of a protein-lipid association rate.

**Figure 2:**
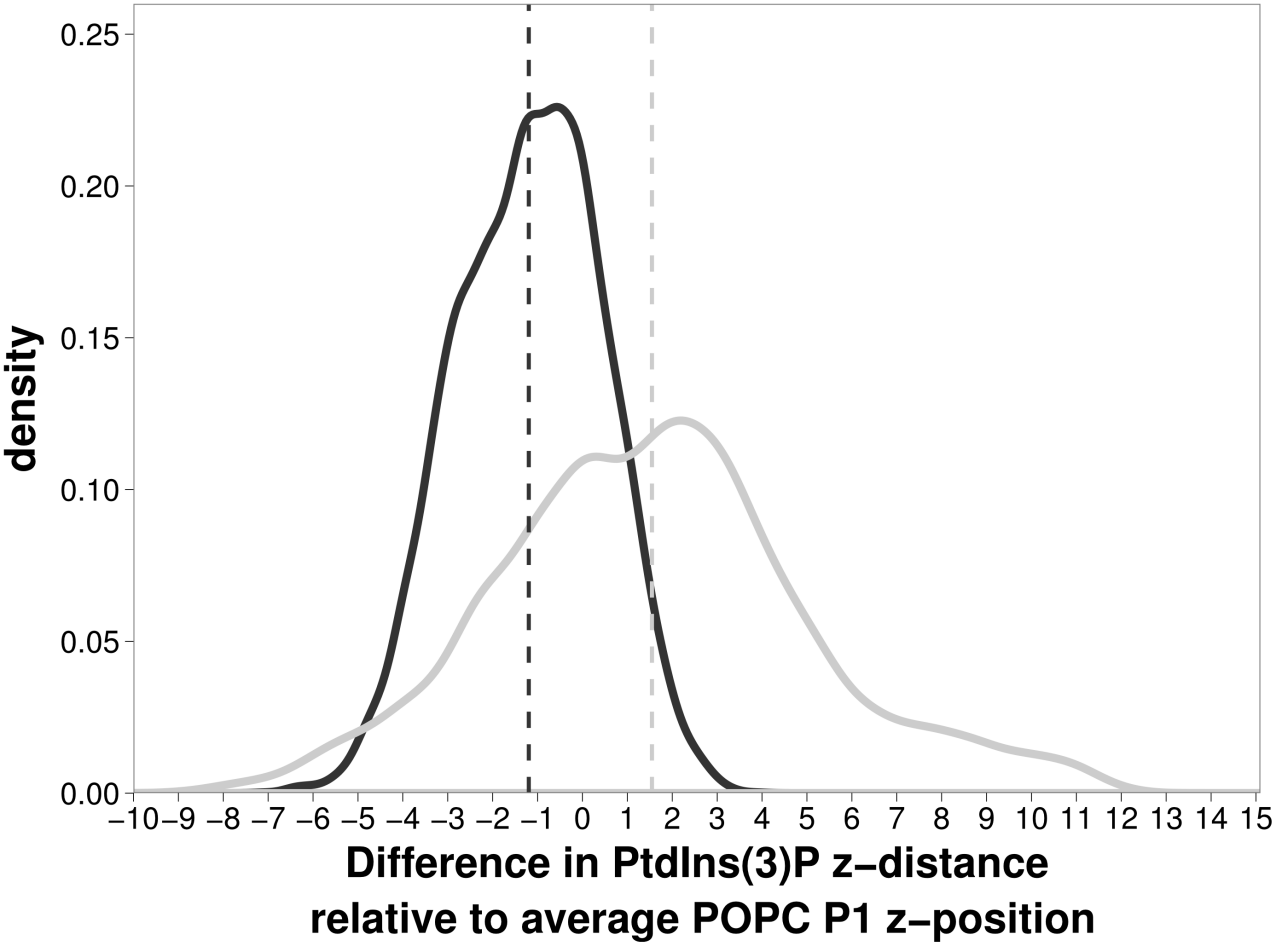
Distribution in the z-direction of the P1 phosphate of PtdIns(3)P relative to the average position of the phosphates of POPC over the time interval from 30 ns to 100 ns. The distribution shows that the difference between the mean distance for the CHARMM c36 simulation (1.5 Å into solvent, dark gray line) and the GAFF simulations (1.2 Å into bilayer, light gray line) is 2.7 Å.

By calculating the z-height of the P1 phosphate of PtdIns(3)P, we found that the PtdIns(3)P molecule protrudes further into the solvent in the simulation using the CHARMM c36 force field compared to the simulation using the GAFF force field. In figure 2, for the two force fields, we show the frequency distribution of the z-heights of the P1 phosphate relative to the average z-height of POPC phosphate atoms further than 15 Å from the PtdIns(3)P P1 phosphate. The mean values of the P1 phosphate position relative to the POPC atoms are statistically significantly different at P<0.1, with the mean being 1.5 Å for CHARMM c36 and −1.2 Å for GAFF. The CHARMM c36 force field simulation has a broader distribution than the GAFF force field. For the CHARMM c36 simulation, more than half of the snapshots show the P1 phosphate to protrude further into the solvent than the average position defined by the POPC phosphates. For the GAFF force field, the converse is true, with more than half of the snapshots showing the P1 phosphate to be more buried in the membrane than the surrounding POPC phosphates. Previous simulations of PtdIns(4,5)P_2_ and PtdIns(3,4,5)P_3_ by Li *et al.* showed that the presence of the phosphatidylinositol lipid caused the formation of a small depression in the z-height of the phosphates of the surrounding POPC lipids, with the PtdIns(4,5)P_2_ and PtdIns(3,4,5)P_3_ protruding slightly into the solvent, 0.1 Å and 0.6 Å respectively in the case of a bilayer containing 72 lipids, with 26 waters/lipid, and 1.1 Å and 1.2 Å respectively in the case of a more hydrated bilayer, 104 waters/lipid [12]. Our simulations were performed with a larger bilayer (608 lipids) and, for CHARMM c36, fall within the error bars calculated by Li *et al.*, indicating agreement between simulations and, furthermore, suggesting that the number of additional phosphates does not significantly affect the z-height of the P1 phosphate. The difference in the observed configuration of the PtdIns(3)P lipid in the bilayer suggests one explanation for the previously discussed difference in the CHARMM c36 isopotential bulge height, which is higher than that observed for the GAFF force field, in concordance with the higher P1 z-height observed for the CHARMM c36 force field.

**Figure 3:**
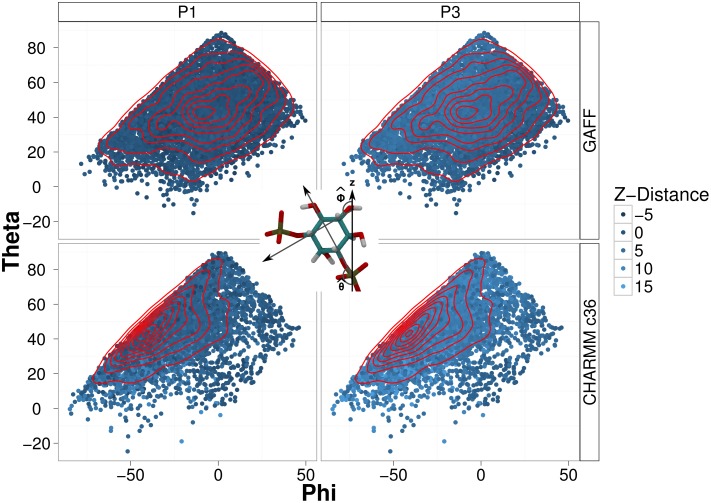
Force field dependent Θ versus Φ behavior with colors representing the height of the P1 and P3 phosphates relative to an axis defined by the membrane (specifically the phosphates of POPC molecules at least 15 Å from the respective PtdIns(3)P phosphates). Relative to the membrane, dark blue colors have a smaller z-distance and light blue colors have a larger z-distance, indicating that the PtdIns(3)P headgroup tends to stick further into the solvent in the CHARMM c36 simulations. The isocontour lines shown in red indicate the density of points plotted. The definitions of Θ (Θ - 90°) (the angle that the vector intersecting the P1 phosphate and the C4 carbon makes with the z-axis) and Φ (Φ - 90°) (the angle that the vector intersecting the C3 and C5 carbons makes with the z-axis) are shown in the central inset.

Differences between the electrostatic potential in the GAFF and CHARMM c36 simulations may arise because the visited regions of the PtdIns(3)P Θ/Φ space differ between the GAFF and CHARMM c36 simulations. The tilt of the PtdIns(3)P headgroup relative to the membrane can be defined by angles Θ and Φ (see Figure 3, inset) and correlates with the z-height of the P1 and P3 phosphates of PtdIns(3)P. Li *et al.* previously used the Θ/Φ angle to determine the most commonly observed conformations of PtdIns(4,5)P_2_ and PtdIns(3,4,5)P_3_ headgroups from their simulations of POPC membranes containing these phosphatidylinositol lipids [12]. Our analysis showed that with the two force fields, the same general region of the Θ/Φ plot was sampled. However, the most probable angles differ between the two force fields. The mean values of the Θ/Φ angles are (44.3°, −26.9°) for the CHARMM c36 simulations and (43.1°, −7.3°) for the GAFF simulations. Simulations by Li *et al.* of PtdIns(4,5)P_2_ and PtdIns(3,4,5)P_3_ in a POPC bilayer showed that some simulations did not sample the full region of Θ/Φ space [12]. Differences in Θ/Φ angle sampling might arise from differences in force field parameters such as the P-O-C-X and X-P-O-C dihedral angles linking the inositol ring to the lipid tail. However, it is difficult to decompose these force field variations and compare directly to the differences in the Θ/Φ angles observed.

**Figure 4:**
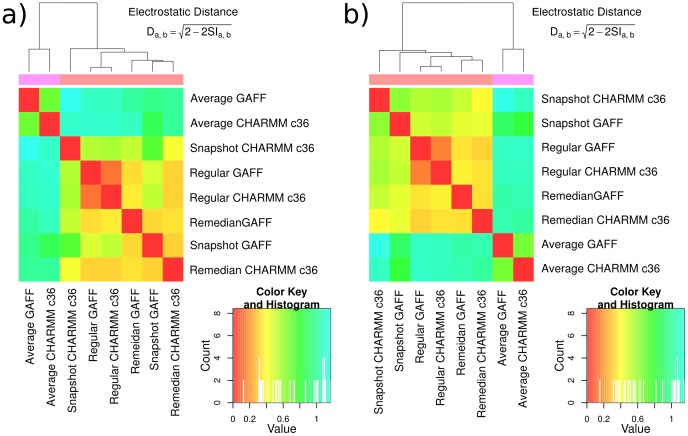
Comparison of the electrostatic potentials of POPC membranes containing a single PtdIns(3)P by PIPSA analysis for a spherical region centered on the P1 atom of PtdIns(3)P and of radius a) 10 Å; and b) 14 Å. The electrostatic distance, D_a,b_, ranges between 0, indicating perfect correlation between potentials, and 2, indicating anti-correlated potentials. See text for details.

We used PIPSA analysis to recapitulate the differences between membrane representations, and to investigate the time-correlations of electrostatic potentials over MD trajectories [19]. The −0.6 kcal/mol/e isopotential bulge is an informative measure of the extent of the electrostatic potential in the vicinity of the PtdIns(3)P lipid, but it cannot give a detailed comparison of the electrostatic environment in 3-dimensions. We used PIPSA to compare the electrostatic potentials within spheres of radius 10 Å and 14 Å centered on the P1 phosphate. In concordance with the isopotential bulge, the PIPSA analysis of the region around PtdIns(3)P shows that the choice of force field makes little difference to the electrostatic potential, whereas the choice of membrane representation can make a large difference. The results presented in figure 4a and 4b show that the most similar electrostatic potential is always observed when the GAFF and CHARMM c36 force field parameters are used to calculate the electrostatic potential for the same bilayer configurations. This observation indicates that the GAFF and CHARMM c36 force fields agree closely on the electrostatic potential generated by the POPC and PtdIns(3)P lipids for a given configuration. Furthermore, the use of PIPSA validates the previous use of the isopotential bulge height as a measure of electrostatic similarity in the context of a lipid membrane. In figure 4a, we see that the electrostatic distance between force fields when using the regular membrane structure is only 0.13 and 0.15 for the 10 and 14 Å spheres. This observation shows that the difference in electrostatic potential due to the force fields is small. Furthermore, we see that the electrostatic potentials for the GAFF and CHARMM c36 MD snapshots have electrostatic distances of 0.73 and 0.63 for the 10 and 14 Å spheres, respectively. Therefore, we infer that the difference in electrostatic potential must be due to differences in the configuration of the bilayer between snapshots. We can further employ PIPSA analysis to identify the time-correlation between a selection of snapshots from one simulation.

**Figure 5:**
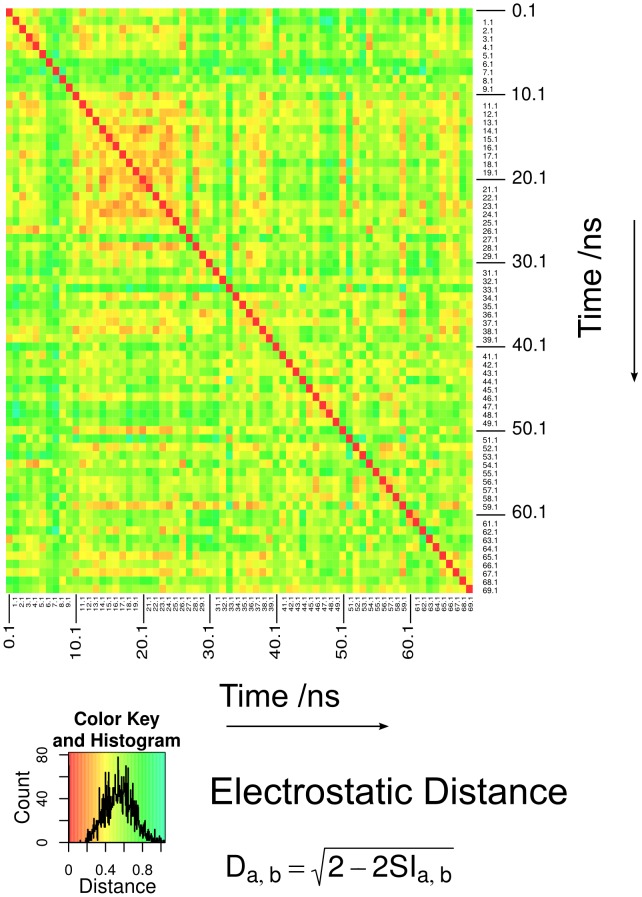
PIPSA analysis of the variation in electrostatic potential between snapshots saved at 1 ns intervals. The heatmap shows the electrostatic distances computed by performing PIPSA for the skin region within a 14 Å sphere centered on the P1 phosphate of PtdIns(3)P for the CHARMM c36 simulations. It can be seen that there is no time-correlation between consecutive snapshots at 1 ns intervals. Furthermore, the histogram of electrostatic distance values shows the range of distances during the simulation.

All-against-all comparison of 70 electrostatic potential grids taken from CHARMM c36 MD simulations every 1 ns in the 30–100 ns interval show that there is no strong time-correlation between electrostatic potentials on this time-scale. The lack of time-correlation can be seen in the PIPSA heatmap in figure 5, where nearby times are rarely observed in nearby clusters. This absence of time correlation was also seen by calculating the autocorrelation function of the electrostatic distance and the time which decays to close to 0 within 1 ns. The distribution of electrostatic distances, as defined by the Hodgkin index as determined by PIPSA, follows a normal distribution (Shapiro-Wilk W = 0.995, p<1×10^−5^) with the mean electrostatic distance between snapshots being 0.55, with standard deviation 0.15.

**Figure 6:**
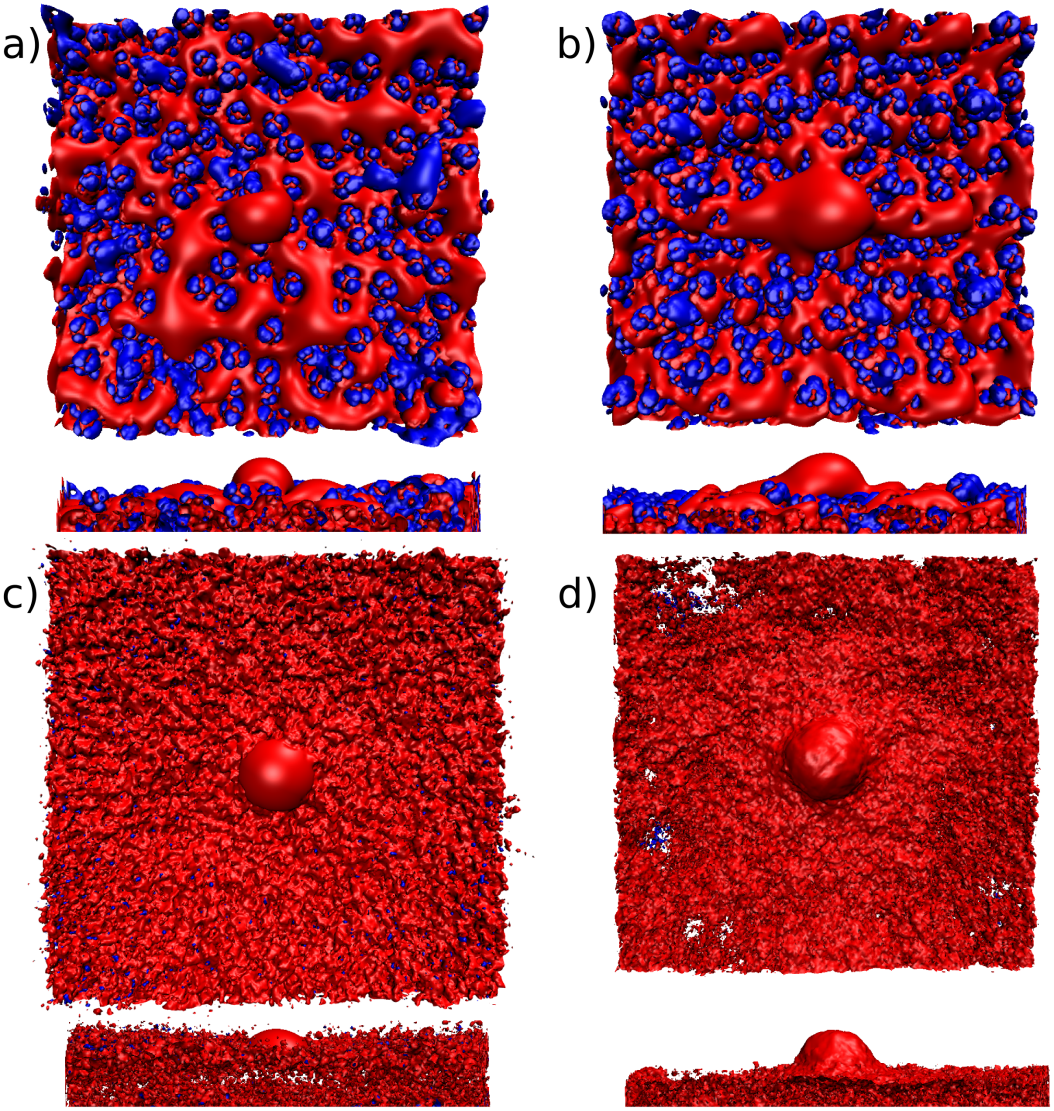
Electrostatic potential of POPC membranes centered on a single PtdIns(3)P. Top and side views are shown of the +/−0.6 kcal/mol/e isopotential contours using the CHARMM c36 force field for a) an MD snapshot; b) a regular membrane; c) the mean potential over snapshots from 30 to 100 ns; d) the remedian potential over snapshots from 30 to 100 ns.

We can compare different ‘non-physical’ representations of the membrane electrostatic potential. The first can be generated using a bilayer constructed from regularly repeating lipids. The second and third rely on averaging techniques using the potential calculated over a range of snapshots from an MD simulation. Previously, in figure 4b, we saw that the difference between the potential grids calculated using the arithmetic mean is large compared to all other methods. The reason for this difference can be seen in Figure 6, where the −0.6 kcal/mol/e electrostatic potential isocontour is shown. While the potential bulge is still observable for the potential calculated using the arithmetic mean over several snapshots, in figure 6c it is clear that a considerable amount of noise is introduced into the grid by using the arithmetic mean. We speculated that the noise was caused by a few grid points with high magnitude electrostatic potentials that bias the mean towards larger values, and therefore used a technique called the remedian to generate an average that is robust to outliers [20]. The result in figure 6d shows the same shape and location of the potential bulge, but the noise visible with the arithmetic mean technique is no longer observed. One difference between the snapshot or the regular membrane and the mean or remedian membrane is the overall negative potential of the latter. For both GAFF and CHARMM c36 force fields, the potential looks similar when comparing the remedian potentials (see figure 6d and Figure S1 d in File S1). Quantitative analysis using PIPSA supports this observation, with the electrostatic distance between remedian potentials 0.35 and 0.34 for 10 and 14 Å radius spheres respectively. The electrostatic distance between mean potentials is 0.68 and 0.67, which indicates more dissimilarity between the two force fields, however, these electrostatic distances are still lower than the distances between other potentials. This suggests that some of the larger electrostatic distances observed for the mean potentials are likely due to noise that is eliminated when comparing potentials calculated using the remedian.

## Conclusions

For a POPC bilayer with a single PtdIns(3)P lipid present, we calculated that a difference between the GAFF and CHARMM c36 force fields is not evident in the case of a ‘regular’ membrane structure when measuring the distance of the −0.6 kcal/mol/e isopotential contour above the P1 phosphate of PtdIns(3)P. Thus the choice of GAFF or CHARMM c36 partial atomic charge parameters to represent the PtdIns(3)P and POPC lipids does not significantly affect the electrostatic potential of this membrane structure. It is clear that, in general, the choice of partial charges may significantly affect the distance of the −0.6 kcal/mol/e isopotential contour. However, given that the majority of molecular dynamics force fields treat electrostatic interactions as Coulombic, we would posit that even using different partial charge calculation methods, such as those used for AMBER or CHARMM respectively, one would hope to identify partial charges that exhibit similar behavior over distances longer than several angstroms.

A difference in the distance of the −0.6 kcal/mol/e isopotential contour above the P1 phosphate of PtdIns(3)P of the two force fields is however evident when taking into account the configurational sampling of the membrane by analyzing the electrostatic behavior over snapshots taken at regular intervals from MD simulations of the membranes. This is further supported by analysis using PIPSA, which has the benefit of comparing the potential over a range of distances rather than just for a single contour [19].

The finding that the choice of GAFF or CHARMM c36 partial charges does not affect the membrane electrostatic potential of a given configuration is echoed by quantitative PIPSA calculations. PIPSA however confirms that the choice of snapshot does have a strong effect on the electrostatic similarity. The differences in the potentials that are observed when using different force fields to generate configurations correlate with further protrusion of the P1 phosphate of PtdIns(3)P from the POPC bilayer. Taken together with the evidence that the choice of force field for partial charge assignment does not affect the potential, we conclude that the configurational sampling of the lipid membrane is very important when considering the observed electrostatic potential. Due to the possibility that full sampling of the PtdIns(3)P Θ/Φ angles has not been acheived, it is not possible to state with confidence that this configurational ensemble is not important. However, the GAFF and CHARMM c36 force fields generate configurations with differing PtdIns(3)P P1 phosphates, suggesting that there is an inherent difference between the force fields.

One issue that this study has not attempted to address is the effect of ions on the electrostatic properties of the bilayer. The effect of sodium chloride ions on bilayers has been previously addressed with molecular dynamics simulations by Böckmann *et al.* [21], where it was shown that when sodium ions bind POPC lipids, the bilayer thickens by approximately 2 Å which has the effect of increasing lipid order parameters. Additionally, the differing treatment of the ion/lipid interaction between force fields may have some effect on the observed electrostatic properties.

The GAFF and CHARMM c36 force fields for lipids have the benefit of being consistent with the AMBER and CHARMM force fields for simulating biological macromolecules as diverse as proteins and nucleic acids, as well as small drug-like molecules [18], [22], [23]. The CHARMM c36 parameters perform robustly and well without the need for a surface tension term, and have been adopted for use in several simulation studies [16]. Since this study was started, the GAFFlipid force field has been developed and it looks to be a promising force field for combination with studies of protein, ligand and lipid membrane association where parameters compatible with the AMBER force fields have already been developed and tested [24]. Furthermore, the Slipids [25]–[27], LIPID11 and LIPID14 [28], [29] force fields have also been developed as new AMBER compatible-models for lipids. We expect that calculations of the electrostatic potential for charged compounds in lipid bilayers with different force fields will show qualitatively similar properties to those we have observed for GAFF and CHARMMc36.

The studies presented in this article suggest that for relatively low concentrations of the PtdIns(3)P lipid (concentrations that are often biologically relevant), an averaged potential may be appropriate for use in Brownian dynamics simulations where the time-scale of interaction is much longer than the correlation time of the fluctuations in electrostatic potential of the membrane. The use of the remedian rather than the median opens up a computationally efficient method to calculate an appropriate potential when many snapshots of interest are available. However, it also suggests that a hybrid method combining an averaged potential formed by the bulk lipids in the bilayer, with atomistic detail provided only for the lipid of interest would be reasonable. It should even be possible to represent this effective background potential using a continuum membrane potential. The knowledge of the electrostatic potential of these types of PtdIns(3)P membranes will allow their further study using BD simulations to investigate the mechanism of membrane recognition by proteins such as FYVE domains.

## Methods

### Electrostatic properties of PtdIns(3)P containing membranes

To assess the effect of the dynamic behavior of the surrounding POPC lipids in bilayers on the electrostatic potential of PtdIns(3)P, we computed the electrostatic potential by numerical solution of the finite-difference non-linear Poisson-Boltzmann equation for 700 snapshots taken every 100 ps from the final 70 ns of the molecular dynamics simulations of PtdIns(3)P containing membranes described in the section *‘Simulation of PtdIns(3)P in a POPC bilayer’*. To allow for a direct comparison between the CHARMM c36 and GAFF force field parameters for POPC, we also computed the electrostatic potential for a bilayer constructed using the Membrane Builder tool in VMD 1.9.1, which places POPC molecules in a bilayer on a hexagonal grid with the hydrophobic tails nearly fully extended [30].

We used UHBD version 6.1 to compute the electrostatic potential of the bilayer [31]. Focusing with two grids was performed. The outer grid had 271×271×222 points and a spacing of 2 Å and encompassed the complete bilayer. The inner grid was centered on the PtdIns(3)P P1 phosphate atom and had 201×201×201 points and a spacing of 0.5 Å. An ionic strength of 100 mM and an ion radius of 2.0 Å were used to allow comparison with previous work [6]. The solvent relative dielectric constant was 80 and the solute relative dielectric constant was 2. The dielectric boundary was defined by the van der Waals surface of the solute. The temperature was set to 296 K.

The electrostatic potential was recorded for a grid of points in the region 0.05 Å above the molecular surface of the bilayer. A python script (see File S2) was written to identify the maximum z-height above the PtdIns(3)P P1 phosphate which is bounded by the −0.6 kcal/mol/e (1 kT/e) isopotential contour. The partial atomic charges and radii from the CHARMM c36 or GAFF force fields were used to calculate the electrostatic potential [16], [18].

### PIPSA analysis of PtdIns(3)P-containing membranes

We used PIPSA v3.0 to perform a quantitative comparison of the electrostatic potentials of a regular bilayer, a snapshot of the bilayer, and two different types of average bilayer structures [19]. The regular membrane was generated using the Membrane Builder tool v1.1 in VMD 1.9.1 [30]. The Membrane Builder tool places POPC lipids with almost fully extended tails on a 2-dimensional hexagonal grid for both the top and bottom bilayer leaflets. The resulting bilayer contained 640 POPC lipids, of which a single lipid in the upper leaflet was replaced with a PtdIns(3)P lipid. Snapshots after 83.5 ns from both the GAFF and the CHARMM c36 simulations were chosen for the PIPSA analysis. Since the grids are all centered on the P1 phosphate of the PtdIns(3)P lipid and each grid is arranged such that the bilayer is perpendicular to the z-axis, coincident grid points can be averaged over a set of grids. The first average bilayer was generated by calculating the arithmetic mean potential at each grid point over all snapshots. Due to the noise observed when using the arithmetic mean, we calculated the median grid for comparison. Due to the large amount of grid data (700 grids of dimension 201^3^), it was necessary to use the remedian as a computationally tractable approximation to the median [20]. The remedian was calculated using 3 arrays of size 9 (suitable for up to 9^3^ = 729 data points) [20]. The python code for computing the remedian is available in File S2. We calculated the similarity between the electrostatic potential for each of these simulated membranes in the skin regions, defined with a 2 Å probe radius and a 3 Å skin thickness, within a sphere centered on the P1 phosphorous atom of PtdIns(3)P. Calculations were performed for two sphere radii, 10 Å and 14 Å. PIPSA analysis was also performed to compute pairwise similarities of the 700 CHARMM c36 simulation snapshots in order to assess the variability between snapshots.

### GAFF parameters for PtdIns(3)P

RESP-A1A charge parameters were calculated using the RED Server version 2 with Gaussian to solve the Hartree-Fock equations [32]–[35]. We supplied the RED Server with a PDB file derived from the previously constructed PtdIns(3)P lipid with the glycerol group capped with two methyl groups (see Figure S2 in File S1). We separately enforced an intra-molecular charge constraint on each of the two methyl groups such that the total charge of each methyl group equaled zero, and constrained each atom in the glycerol group to the exact charge parameters of the POPC lipid derived by Jójárt and Martinek [15]. Topology files for Gromacs were generated using the acpype front-end to Antechamber.

### CHARMM parameters for PtdIns(3)P

CHARMM parameters for PtdIns(3)P were created by analogy with existing parameters for the CHARMM c36 POPC lipids and the CHARMM carbohydrate parameters (full details available in the supporting information section *‘Deriving CHARMM c36 compatible parameters for PtdIns(3)P for use in Gromacs.’* in File S1) [16], [36]. We used the CC3161, HCA1, OC311, and HCP1 atom types from CHARMM Carbo [36]. To create a phosphatidyl-inositol compound, we created a bond between OC311 (from CHARMM Carbo) and PL (from CHARMM c36). The resulting compound had a net charge of −0.95e rather than −1e as the two groups originally had, so we adjusted the charges of atom types O2L from −0.78 to −0.80e and OSLP from −0.57 to −0.58e (see Figure S3 in File S1). To add the phosphate at position three on the inositol ring, we used the phosphate atom types PL and O2L from POPC. Here, we altered the charge of PL from +1.50 to +1.52e, and O2L from −0.78 to −1.02e to create a net charge of the group of −2e (see Figure S3 in File S1). The total charge of the PtdIns(3)P lipid was again −3e.

### Simulation of PtdIns(3)P in a POPC bilayer

A large pre-equilibrated POPC bilayer from a previous study, by Cojocaru *et al.*, containing 608 POPC lipids and 50362 waters was taken and one of the POPC lipids was substituted by a PtdIns(3)P phospholipid [37]. The first POPC molecule listed in the PDB file was mutated to a PtdIns(3)P using the Maestro software to build the crystallographic coordinates of an Ins(1,3)P_2_ molecule from the PDB coordinate file 1JOC onto the POPC phosphate group. The Ins(1,3)P_2_ molecule had been previously protonated using the Ligprep functionality of Maestro to give a total charge of −3 e for the PtdIns(3)P molecule. This bilayer was then simulated using the same procedures as described below for the GAFF and CHARMM c36 simulations. Parameters for PtdIns(3)P suitable for use with GAFF and CHARMM c36 are described in the following sections.

Simulations of pure POPC bilayers were performed using the Gromacs 4.5.3 software [38]. Our simulation protocols followed the published protocols for POPC lipid simulations using the GAFF and CHARMM c36 force fields as closely as possible. However, Jójárt and Martinek used AMBER 8 and NAMD 2.6 to perform their simulations, and Klauda *et al.* used CHARMM and NAMD to perform their simulations [15], [16], [39], [40]. For both systems, no counter ions were added, since this follows the original procedure described by Jójárt and Martinek [15] In cases where we could not follow the published protocol exactly, we chose parameters that are as close to the published parameters as possible. Furthermore, we performed simulations to compare the results of pure POPC simulations using Gromacs. The area per lipid of the pure POPC bilayers was calculated as 61.0±1.0 Å^2^, which is close to the 59.2±0.5 Å^2^ range calculated by Jójárt and Martinek [15], see Figure S4 in File S1. In the case of CHARMM simulations of POPC lipids, this test has been performed previously by Piggot *et al.* [41], and our own validation calculations, 63.0±0.8 Å^2^ are close to the 64.7±0.2 Å^2^ calculated by Klauda *et al.* [16], and certainly fall within the relatively large experimental uncertainty, see Figure S5 and Table T1 both in File S1. We specify the parameters used for each simulation in the section below. Input files used for the simulations are given in the supporting information and have been submitted to the Lipidbook website. Analysis of standard properties of the bilayers, such as area per lipid are given in File S1.

### Preparation and simulation of a POPC bilayer

We used the previously described system of 608 POPC lipids and 50362 TIP3P waters. In both cases, input files are available in File S6. The following specific systems and parameters were used for the different force fields:

#### GAFF

A Berendsen barostat with a time constant of 1 ps and an isothermal compressibility of 4.5×10^−5^ bar nm^−1^ was used to maintain a pressure of 1 bar in the z-direction, and a surface-tension of 600 bar nm. To enable the target temperature of 296 K to be reached, a simulation was performed for 500 ps in which neighbor lists were updated using a grid-based search procedure every 10 integration steps. The neighbor list cutoff distance was 1.0 nm and short-range electrostatic and van der Waals cutoffs were 1.0 nm by default. PME was used to treat long-range electrostatics, with PME order 6, and Fourier spacing 0.15 nm. Four simulations of 100 ns were performed using short-range electrostatic and van der Waals cutoffs of 1.0 nm, 1.2 nm, 1.4 nm, and 1.6 nm.

#### CHARMM c36

The same procedure as for the GAFF simulations was used to perform the 500 ps temperature equilibration. Neighbor search lists remained at 1.0 nm, but the long range list was extended to 1.4 nm. Electrostatic interactions were calculated with PME with a cut off at 1.0 nm, the PME order was 6 and the Fourier spacing 0.15 nm. Van der Waals interactions were switched in the range 0.8 nm to 1.2 nm. Each simulation was 100 ns long and the Nose-Hoover thermostat was used to couple lipids and water separately using a reference temperature of 296 K and a time constant of 1.0 ps [42], [43]. The Parrinello-Rahman barostat was applied in the x-y plane and in the z-direction using a time constant of 5.0 ps, an isothermal compressibility of 4.5×10^−5^ bar nm^−1^ and a reference pressure of 1.0 bar [44].

## Supporting Information

File S1 **Summary of additional supporting information, description of CHARMM c36 compatible PtdIns(3)P parameter development, supporting figures S1–S5 and supporting table T1, Appendix 1 containing parameterization of PtdIns(3)P for compatibility with CHARMM c36 in Gromacs.**
(PDF)

File S2 **Python script for calculation of isopotential height, python script for calculation of average and remedian electrostatic potentials, GAFF and CHARMM Gromacs input files (mdp, pdb, top, itp) for PtdIns(3)P and POPC (GAFF), POPC parameters for CHARMM are available from the Gromacs website).**
(ZIP)

File S3 **Bilayer remedian electrostatic grids for CHARMM and GAFF force fields in UHBD binary format.**
(ZIP)

File S4 **Bilayer average electrostatic grids for CHARMM and GAFF force fields in UHBD binary format.**
(ZIP)

File S5 **Regular bilayer electrostatic grids for CHARMM and GAFF force fields in UHBD binary format.**
(ZIP)

File S6 **GAFF and CHARMM bilayer snapshot electrostatic grids in UHBD binary format, including UHBD input files for membrane electrostatic calculations.**
(ZIP)
