# Supplementary material for: On Calculation of the Electrostatic Potential of a Phosphatidylinositol Phosphate-Containing Phosphatidylcholine Lipid Membrane Accounting for Membrane Dynamics
Source: PLoS One. 2014 Aug 20;9(8):e104778. doi: 10.1371/journal.pone.0104778 (PMC4139306; doi:10.1371/journal.pone.0104778)
Supplement: File S1 — Summary of additional supporting information, description of CHARMM c36 compatible PtdIns(3)P parameter development, supporting figures S1–S5 and supporting table T1, Appendix 1 containing parameterization of PtdIns(3)P for compatibility with CHARMM c36 in Gromacs. (PDF) [file pone.0104778.s001.pdf]

Supporting information for "On calculation of the electrostatic potential of a phosphatidylinositol phosphate-containing phosphatidylcholine lipid membrane accounting for membrane dynamics"

Jonathan C. Fuller[1], Michael Martinez[1], Rebecca C. Wade[1,2]**

[1]Heidelberg Institute for Theoretical Studies, Schloss-Wolfsbrunnenweg 35, 69118 Heidelberg, Germany

[2]Center for Molecular Biology (ZMBH), Heidelberg University, Germany

*Corresponding authors: JCF jonathan.fuller@h-its.org and RCW rebecca.wade@h-its.org

Additional supporting information is available as zip files containing:

- GAFF parameters for GROMACS (popc_gaff_jojartandmartinek_GMX.itp)
- PtdIns(3)P Gromacs files (ptdins3p_GAFF_GMX.itp, ptdins3p_CHARMM_GMX.top)
- PDB file starting coordinates for PtdIns(3)P (popc_ptd_solv.pdb)
- Isocontour python script, the script calculates the isopotential height of the electrostatic potential from the lipid bilayer: isopotential_height.py
- Remedian python scripts, the script calculates the remedian potential for a set of electrostatic potential grids: remedian.py
- Average python scripts, the script calculates the average potential for a set of electrostatic potential grids: average.py
- Input files for PtdIns(3)P simulations (gaffptd.mdp, charmmptd.mdp)
- Electrostatic potential calculation scripts (AlignSurfaceOnXYplane_script.py, ptd_charge.py, qtable_popc_charmm.dat, qtable_popc_charmm.dat, qtable_withLIP_SIT_CG, uhbd_focus_test_excl_repCG.in, uhbd_focus_test_excl_repCG_charmm.in)
- All bilayer electrostatic grids used for generating figure 4 (Bilayer electrostatic grids directory)

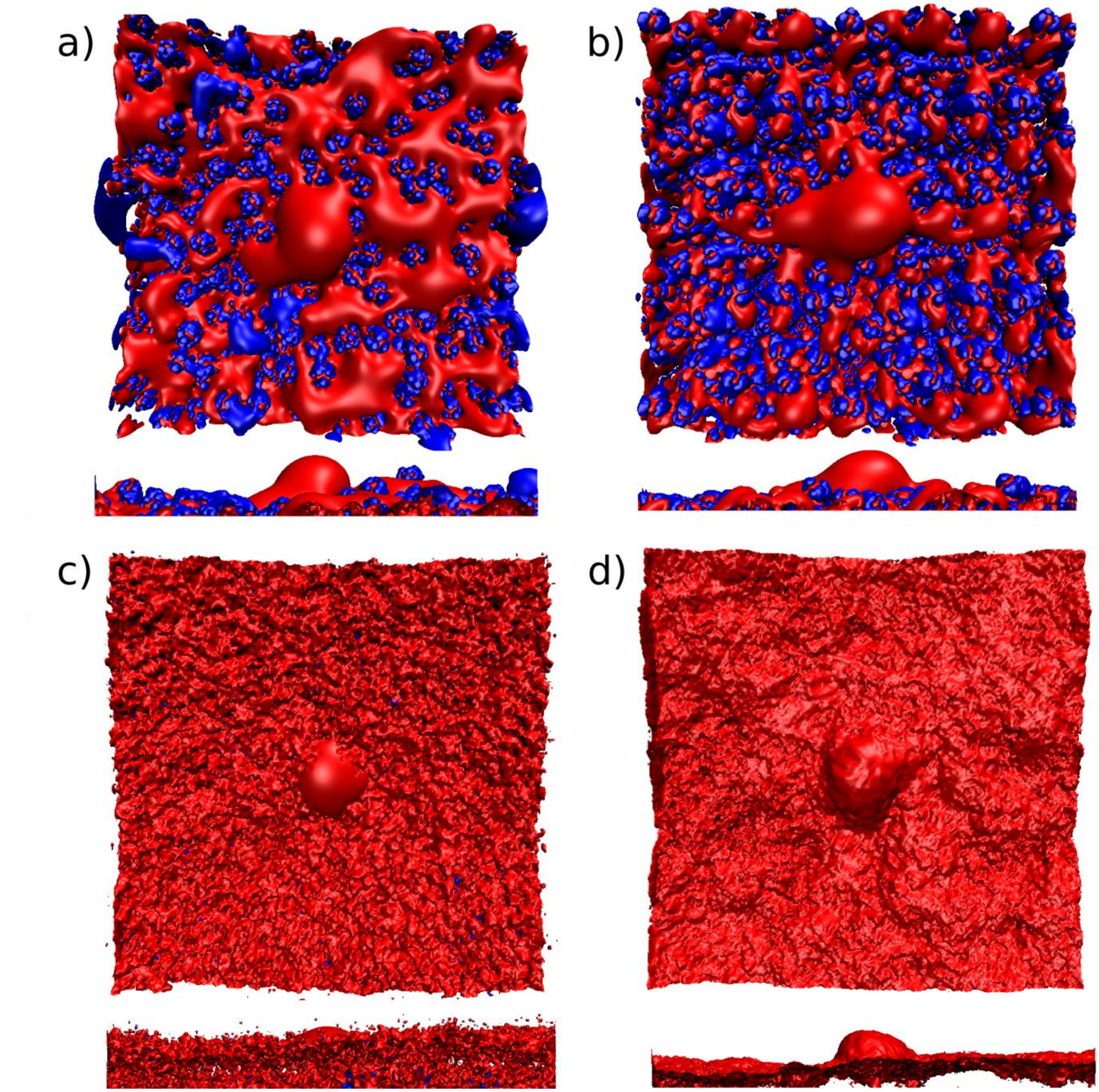

Supporting Figure S1: Electrostatic potential of POPC membranes centered on a single PtdIns(3)P, top and side views of the +/- 0.6 kcal/mol/e contours using the GAFF force field for a) an MD snapshot; b) a regular membrane; c) the mean potential over snapshots from 30-100 ns; d) the remedian potential over snapshots from 30-100 ns.

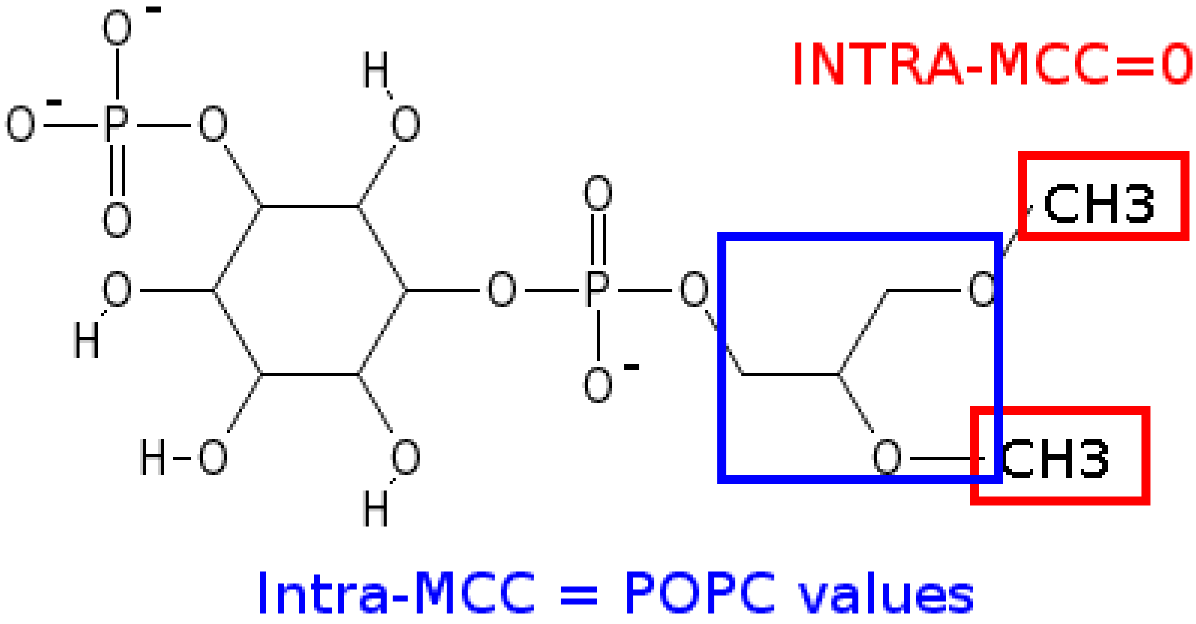

Supporting Figure S2: Derivation of partial atomic charges for the PtdIns(3)P headgroup using the RED server. The groups to which charge constraints were applied are boxed.[1] For the methyl groups (red boxes), the total charge of the group was constrained to zero, and for the glycerol group (blue box), each partial atomic charge was constrained to its value in the parameters for POPC derived by Jójárt and Martinek.[2]

Deriving CHARMM c36 compatible parameters for PtdIns(3)P for use in Gromacs.

No parameters for PtdIns(3)P that are compatible with the CHARMM c36 force field are provided with Gromacs. Our parameterization followed a strategy close to that of Li *et al*. For the PtdIns(3)P lipid tail, we used the parameters for POPC in the CHARMM c36 force field[3] (files from the Gromacs mailing list).[4] A hybrid of the CHARMM c36 force field[3] and the CHARMM carbohydrate force field[5] was created by taking lipid tail parameters from CHARMM c36 and lipid headgroup parameters from CHARMM carbohydrate. In the case where differing atom types were available for an atom, the best matching atom type was selected (see supporting figure S2).[5] After this procedure, several atoms were still untyped or had contradictory charges. Therefore, a choice was made as to which atom type or charge value to use. In the latter case, appropriate charges were chosen so that the deviation from the net charge was minimized (see supporting figure S2). In the former case, appropriate atom types were selected that minimized the number of new bonded terms required (see supporting figure S2). To implement the CHARMM carbohydrate force field in Gromacs, scripts written by Mark Abraham (available on the Gromacs website) were used to convert the CHARMM carbohydrate parameters to Gromacs format.[6] To implement the force field in Gromacs, the missing bond, angle and dihedral terms were substituted with the appropriate values and the 1-4 interactions were explicitly calculated. Details are in Appendix 1 of this document.

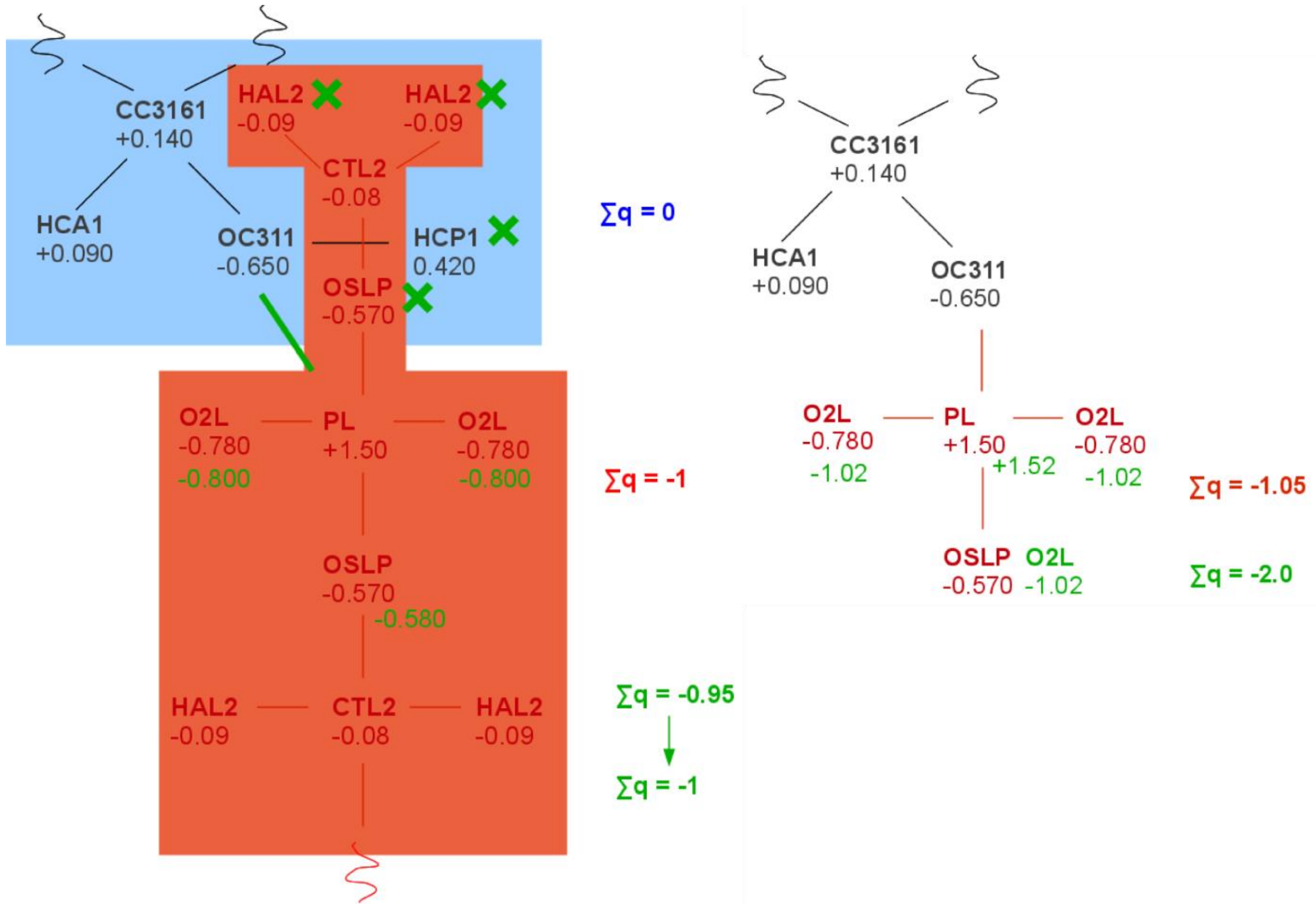

Supporting Figure S3: Assignment of atom types and partial atomic charges for the PtdIns(3)P lipid for use with the CHARMM c36 force field. Left: the P1 phosphate bound to the glycerol group of the lipid tails and the inositol ring. Right: the P3 phosphate bound to the inositol ring at the C3 carbon position. A net charge on the PtdIns(3)P of -3e is assumed, as for the GAFF force field.

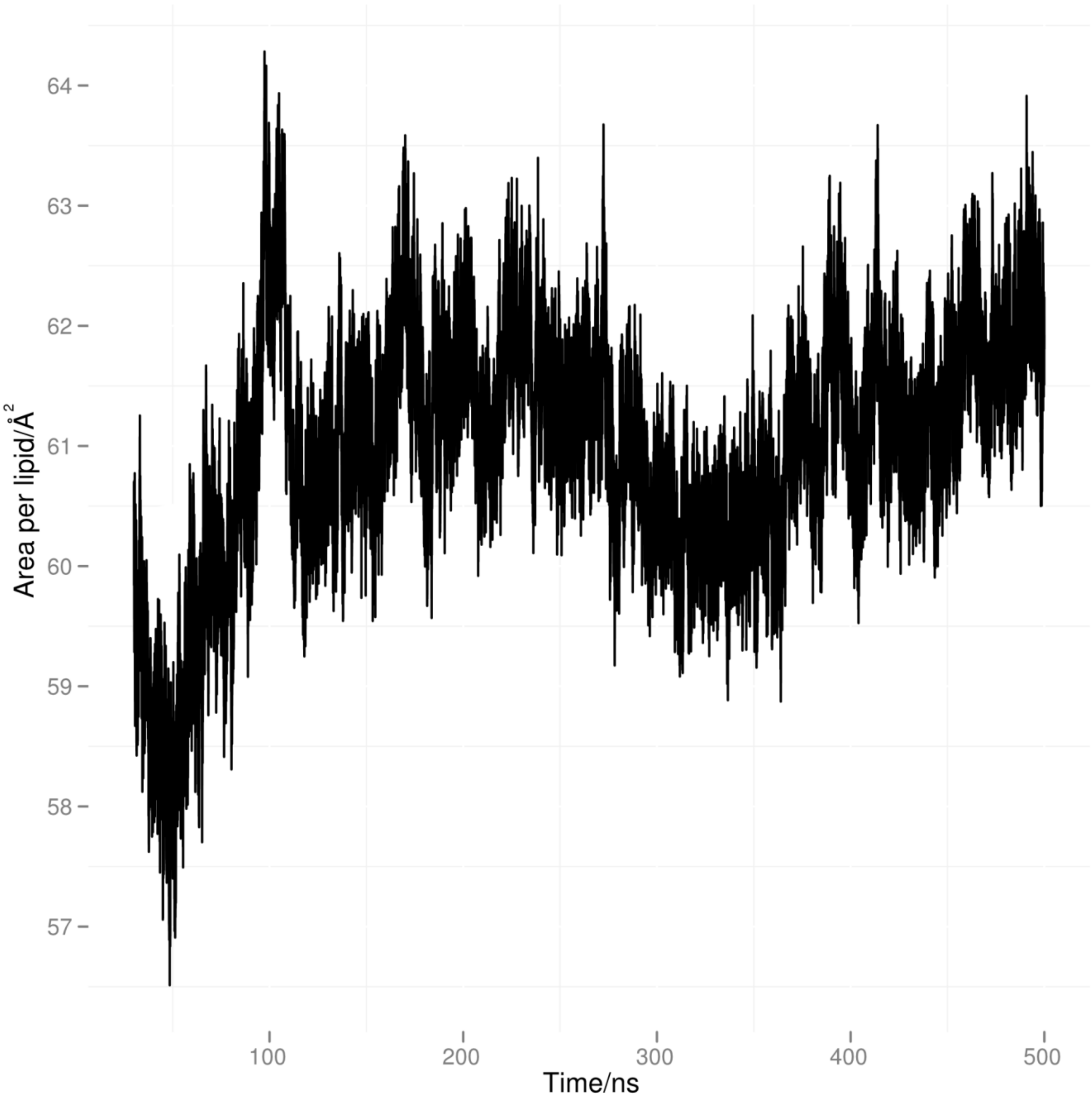

Supporting Figure S4: Area per lipid as a function of time over 500 ns total simulation time, of a POPC bilayer performed at 296 K in Gromacs 4.5.3 with the GAFF force field parameters reported by Jójárt and Martinek.[2]

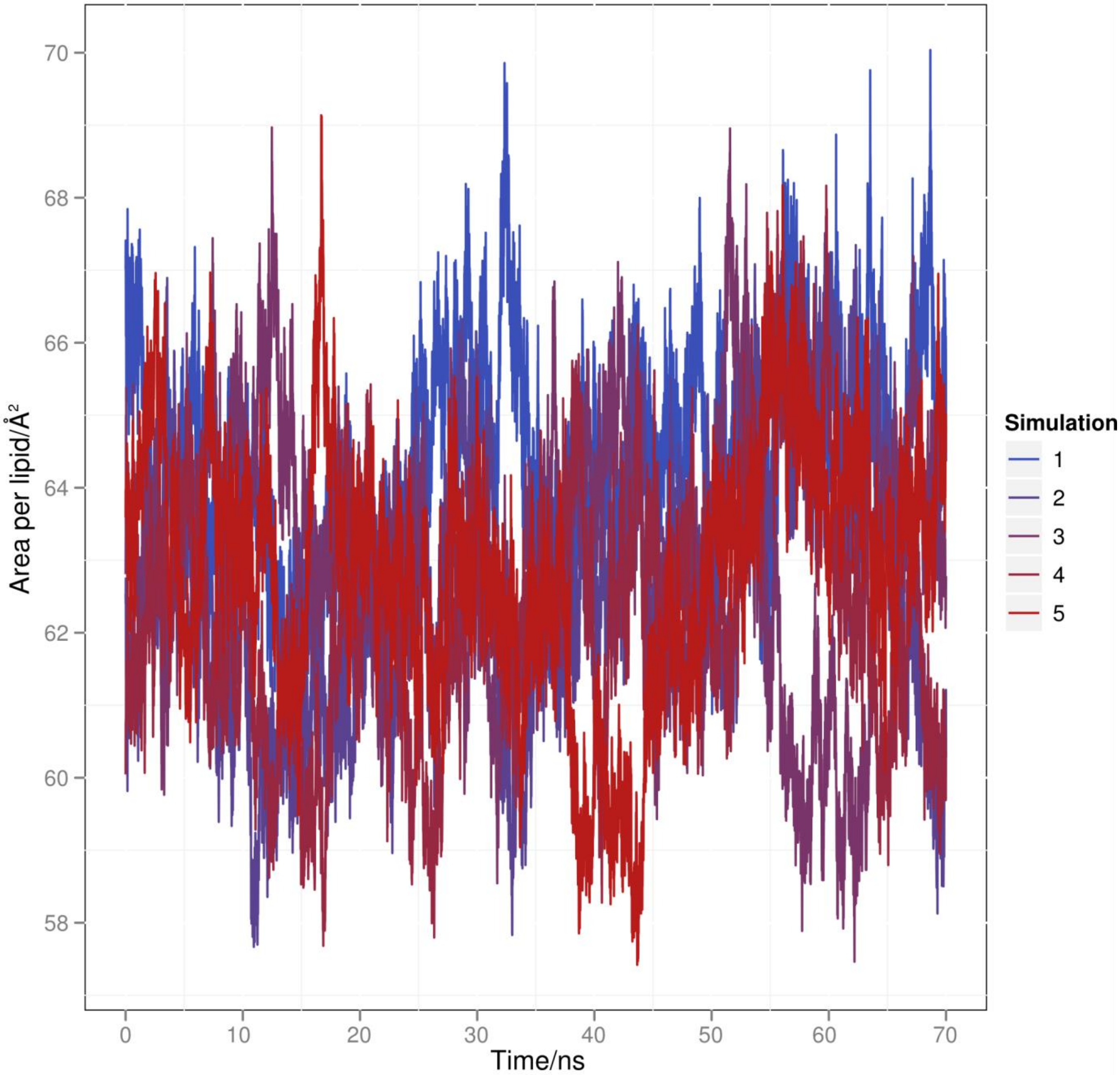

Supporting Figure S5: Area per lipid as a function of time for five simulations of a POPC bilayer performed at 296 K in Gromacs 4.5.3 with the CHARMM c36 force field parameters reported by Klauda et al.[3] All the simulations were started from the coordinates of the 72 POPC lipid bilayer reported by Klauda *et al*.[3]

**Simulation**	**Area per lipid/Å^2**	**Thickness/Å**	**MSD 1×10^8 (Å^2/s)**
Simulation 1[§]	64.3 ± (1.5)	38.8 ± (0.7)	12.0 ± (1.0)
Simulation 2	62.6 ± (1.8)	39.6 ± (0.9)	9.2 ± (0.9)
Simulation 3	62.9 ± (1.8)	39.5 ± (1.0)	13.5 ± (3.3)
Simulation 4	62.3 ± (1.7)	39.8 ± (0.9)	10.1 ± (1.7)
Simulation 5	62.9 ± (1.8)	39.5 ± (0.9)	13.5 ± (3.9)
Average	63.0 ± (0.8)	39.4 ± (0.4)	11.7 ± (2.8)

Supporting Table T1: Area per lipid, bilayer thickness (P-P distance) and diffusion constant (MSD) computed from 5 simulations of the 72 POPC lipid bilayer using the CHARMM c36 force field parameters reported by Klauda *et al.*[3]

References

(1) Vanquelef, E.; Simon, S.; Marquant, G.; Garcia, E.; Klimerak, G.; Delepine, J. C.; Cieplak, P.; Dupradeau, F.-Y. *Nucleic Acids Res.* **2011**, *39*, W511–7.

(2) Jójárt, B.; Martinek, T. A. *J. Comput. Chem.* **2007**, *28*, 2051–8.

(3) Klauda, J. B.; Venable, R. M.; Freites, J. A.; O'Connor, J. W.; Tobias, D. J.; Mondragon-Ramirez, C.; Vorobyov, I.; MacKerell, A. D.; Pastor, R. W. *J. Phys. Chem. B* **2010**, *114*, 7830–43.

(4) Li, Z.; Venable, R. M.; Rogers, L. A.; Murray, D.; Pastor, R. W. *Biophys. J.* **2009**, *97*, 155–63.

(5) Hatcher, E.; Guvench, O.; Mackerell, A. D. *J. Chem. Theory Comput.* **2009**, *5*, 1315–1327.

(6) Hess, B.; Kutzner, C.; Spoel, D. van der; Lindahl, E. *J. Chem. Theory Comput.* **2008**, *4*, 435–447.

(7) Mallajosyula, S. S.; Guvench, O.; Hatcher, E.; MacKerell, A. D. *J. Chem. Theory Comput.* **2012**, *8*, 759–776.

Appendix 1: Parameterization of PtdIns(3)P for compatibility with CHARMM c36 in Gromacs.

Bonded terms.

In the case of the bonded terms, we used the lipid terms rather than the carbohydrate terms from the force field,[3] since there was no phosphate atom parameterized in the carbohydrate force field. New phosphate parameters have been made available recently.[7]

Bonds.
Only one new bonded term was required: OC311-PL, which we assumed has the same parameters as PL-OSLP.

Added to bonded.itp
```
; Added OC311-PL
OC311    PL      1       0.16    225936.0
```

Bond Angles.
CC3161-OC311-PL input parameters chosen by analogy to: CTL3-OSLP-PL (we assumed that CC3161 has approximately tetrahedral geometry).
OC311-PL-O2L input parameters chosen by analogy to: OSLP-PL-O2L
OC311-PL-OSLP input parameters chosen by analogy to: OSLP-PL-OSLP

Added to bonded.itp
```
; CC3161-OC311-PL; OC311-PL-O2L; OC311-PL-OSLP
CC3161  OC311   PL      5     120.0   167.36   0.233   29288.0 ; New
OC311   PL      O2L     5     111.6   827.5952 0.0     0.0     ; New
OC311   PL      OSLP    5     104.3   669.44   0.0     0.0     ; New
```

Dihedral angles.
CC3161-OC311-PL-OSLP input parameters chosen by analogy to: CTL3-OSLP-PL-OSLP
CC3161-OC311-PL-O2L input parameters chosen by analogy to: CTL3-OSLP-PL-O2L
HCA1-CC3631-OC311-PL input parameters chosen by analogy to: PL OSLP CTL2 CTL2
CC3161-CC3161-OC311-PL input parameters chosen by analogy to: CTL3-CTL3-OSLP-PL

Added to bonded.itp
```
; CC3161-OC311-PL-OSLP; CC3161-OC311-PL-O2L ; HCA1-CC3631-OC311-PL;
CC3161-CC3161-OC311-PL
OSLP    PL      OC311   CC3161  9       180.00  5.0208   1 ; New
OSLP    PL      OC311   CC3161  9       180.00  0.4184   2 ; New
OSLP    PL      OC311   CC3161  9       180.00  0.4184   3 ; New
O2L     PL      OC311   CC3161  9       0.00    0.4184   3 ; New
PL      OC311   CC3161  CC3161  9       0.00    1.702888 2 ; New
PL      OC311   CC3161  CC3161  9       180.00  1.008344 1 ; New
PL      OC311   CC3161  HCA1    9       0.00    1.702888 2 ; New
PL      OC311   CC3161  HCA1    9       180.00  1.008344 1 ; New
```

Improper torsions.

No impropers added.

Nonbonded terms.

Electrostatics.
See Supporting Figure S2 for partial atomic charges.

Lennard Jones.
We use the values specified for the atom type in the files Charmm36 or Charmm carbohydrate.

1-4 interactions.
Since the 1-4 interactions are defined by the modified (usually Carbon) LJ parameters combined with the standard parameters for the second atom, all parameters are defined unambiguously. However, in the case of 1-4 interactions between the Charmm36 and Charmm carbohydrate atom types, they are undefined. Here we calculate them from the parameter files for the atom types specified below, using the relationship (where R_{min} is the sum of each $R_{min}/2$ value):

$$\sigma = \frac{1}{10}\frac{R_{min}}{2^{\frac{1}{6}}}$$

CC3161 – PL
CC3161 – HCP1
CC3161 – HCA1
CC3161 – OC311
CC3161 – CC3161
CC3161 – O2L
CC3161 – OSLP
CTL2 – OC311 (CTL2 has same 1-4 as CTL3 as CC3161, thus equals CC3161-OC311)

Added to nonbonded.itp

```
; New 1-4 Params calculated
CC3161    PL      1       0.360813980846  0.320014464673
CC3161    OC311   1       0.32651438  0.183381431 ; Not in C27
CC3161    HCA1    1       0.288651184678  0.0887560432
CC3161    OSLP    1       0.316269044940  0.132309697301 ; Not in C27
CC3161    CC3161  1       0.338541512893  0.04184
CC3161    OSLP    1       0.316269044940  0.132309697301 ; Not in C27
CC3161    O2L     1       0.320723538531  0.144938011577
CC3161    HCP1    1       0.189271432669  0.0897368027066 ; From CTL3
HCL (is the same)
CTL2      OC311   1       0.32651438  0.183381431; equivalent to
CC3161 - OC311
```

New atoms in the atomtypes.atp file:

Added to atomtypes.atp

```
### (for PTD)
CC3161    12.011000 ; Carbon in an inositol ring
OC311     15.999400 ; Inositol oxygen
HCA1      1.008000 ; Inositol hydrogen
HCP1      1.008000 ; Inositol hydrogen bound to oxygen
```
